# Therapeutic Effects and Mechanisms of Action of Garlic (*Allium sativum*) on Nonalcoholic Fatty Liver Disease: A Comprehensive Systematic Literature Review

**DOI:** 10.1155/2022/6960211

**Published:** 2022-10-06

**Authors:** Sanaz Pourreza, Pouria Sefidmooye Azar, Sarvin Sanaie, Nooshin Noshadi, Saeedeh Jalali, Hamid Reza Niazkar, Arash karimi, Mahdi Vajdi

**Affiliations:** ^1^Department of Community Nutrition, School of Nutritional Sciences and Dietetics, University of Medical Sciences, Tehran, Iran; ^2^Nutrition Research Center, Faculty of Nutrition and Food Sciences, Tabriz University of Medical Sciences, Tabriz, Iran; ^3^Research Center for Integrative Medicine in Aging, Aging Research Institute, Tabriz University of Medical Sciences, Tabriz, Iran; ^4^Nutrition Research Center, Department of Clinical Nutrition, School of Nutrition and Food Sciences, Tabriz University of Medical Sciences, Tabriz, Iran; ^5^Department of Clinical Nutrition, School of Nutrition and Food Science, University of Medical Sciences, Shiraz, Iran; ^6^Breast Diseases Research Center, Shiraz University of Medical Sciences, Shiraz, Iran; ^7^Student Research Committee, Department of Clinical Nutrition, School of Nutrition and Food Science, Isfahan University of Medical Sciences, Isfahan, Iran

## Abstract

Nonalcoholic fatty liver disease (NAFLD) is globally the leading cause of hepatic dysfunction. Garlic has many physiological benefits, including anti-inflammatory, antioxidant, anticancer, lipid-lowering, and antidiabetes effects. The present study aimed to systematically review the effects of garlic (*Allium sativum*) and its mechanisms of function in managing NAFLD and its associated complications. The guidelines of the Preferred Reporting Items for Systematic Review and Meta-Analysis (PRISMA) statements were applied to perform the study (CRD42021289348). The Scopus, Embase, Web of Science, Cochrane PubMed, and Google Scholar databases were searched until February 2022. According to the inclusion criteria, finally, 12 studies were entered into the study. The evidence provided in the study revealed that garlic could regulate the development of NAFLD via several mechanisms of action, such as lowering body weight, modulating lipid and glucose metabolism, and reducing inflammation and oxidative stress (OS). Overall, the beneficial effects of garlic in the treatment of NAFLD make it a potential therapeutic and efficient agent in managing NAFLD and its related risk factors. There is an insufficient number of clinical trials addressing the effects of garlic in humans; therefore, conducting more human research in the future is recommended.

## 1. Introduction

Nonalcoholic fatty liver disease (NAFLD) consists of a wide range of disorders, from simple fatty liver to nonalcoholic steatohepatitis (NASH) and progressive fibrosis that eventually leads to cirrhosis [1]. NAFLD is the most frequent reason for liver diseases globally, with a prevalence estimation of 25% to 45%, and it is on the rise in tandem with obesity and diabetes [2, 3]. In 1998, for the first time, it was suggested that NAFLD could be considered a disease with a “two-hit” process [4]. In the first hit, a high-fat diet (HFD), obesity, sedentary lifestyle, and insulin resistance (IR) result in an increase in lipid accumulation in the liver cells [5]. Oxidative stress (OS) is one of the main factors contributing to NAFLD progression in the second hit [6, 7]. The “two-hit” model was revised in 2010 with a “multiple parallel hit” model. In the revised model, fat accumulation in the liver and IR, multiple simultaneous changes lead to an imbalance between antilipotoxic protection systems in the liver and the production of free radicals, endoplasmic stress, decrease in mitochondrial content, disruption of mitochondrial B-oxidation, OS, overproduction of reactive nitrogen and oxygen species (RNS/ROS), and death of liver cells [8–10]. Even though obesity, especially central obesity, is a well-known risk factor for NAFLD, it has also been reported in slim people (BMI <30 kg/m2) [11]. Furthermore, IR and metabolic syndrome (MetS), a group of cardiovascular risk factors that include visceral obesity, hypertension, dyslipidemia, and glucose intolerance, are the pathophysiological elements in NAFLD [12]. For NAFLD treatment, lifestyle modifications are commonly recommended, including eating a healthy diet, losing weight, and engaging in regular physical activity. However, this strategy alone cannot reduce the growing prevalence of the disease [13]. Many studies have shown that natural and herbal compounds effectively reduce the symptoms of diseases, such as neurodegenerative disease, diabetes, neurological diseases, and fatty liver [14–21]. Plant-derived natural substances such as phenolic compounds, anthocyanin, wogonin, glycyrrhizin, green tea, coffee, garlic, soybean, and fenugreek might improve NAFLD [22–24]. Garlic (*Allium sativum* L.) has been used as a natural medicine for centuries and is consumed in many countries today [25]. Allicin, ajoene, diallyl disulfide, *S*-allylcysteine (SAC), *S*-methylcysteine sulfoxide, and SAC sulfoxide are some bioactive compounds found in garlic, and the therapeutic benefits of garlic can be attributed to them [26, 27]. Garlic has a wide range of physiological benefits, including anti-inflammatory, antioxidant, anticancer, lipid-lowering, and antidiabetic effects [28]. Evidence demonstrates that garlic consumption can prevent atherosclerosis, diminish blood pressure, boost fibrinolytic activity, and inhibit platelet aggregation [29]. Furthermore, it has recently been proposed that garlic has a favorable effect on liver enzymes such as gamma-glutamyltransferase (GGT), alanine transaminase (ALT), and fatty liver [30, 31]. Clinical and experimental research indicated that garlic could affect IR and obesity, which are connected to NAFLD pathogenesis [32, 33]. Thus, garlic may positively affect the development of NAFLD and its relevant complications [34]. As a result, this systematic review aimed to evaluate garlic's therapeutic efficacy in managing NAFLD and its related risk factors and putative mechanisms of action.

## 2. Methods

The Preferred Reporting Items for Systematic Reviews and Meta-Analyses (PRISMA) checklist was used to conduct this systematic review. The protocol for this review was registered in the PROSPERO database (CRD42021289348).

### 2.1. Search Strategy

An online search of Scopus, Embase, Web of Science, Cochrane PubMed, and Google Scholar databases was conducted by two authors to find relevant English-language articles published until February 2023. We searched for articles using MeSH and non-MeSH terms, including (“garlic” [MESH] or “raw garlic” [TIAB] or “allium sativum” [MESH] or “fermented garlic” [TIAB] or “non-fermented garlic” [TIAB] or “black garlic” [MESH] or “diallyl disulfide” [TIAB]) and (“non-alcoholic fatty liver disease” [MESH] or “fatty liver” [TIAB] or “NAFLD” [MESH] or “liver fibrosis” [TIAB] or “NASH” [TIAB] or “non-alcoholic steatohepatitis” [TIAB] or “hepatic steatosis” [TIAB] or “insulin resistance” [TIAB] or “inflammation” [MESH] or “oxidative stress” [MESH] or “obesity” [MESH] or “BMI” [TIAB], “fat mass” [TIAB] or “dyslipidaemia” [TIAB] or “free fatty acids” [TIAB]) or “Glycaemic indices” [MESH (the methods of searching in different databases are attached to Supplementary Table 1). All studies were transferred to EndNote software (version X8, for Windows, Thomson Reuters). Additionally, we cross-checked the references of the retrieved studies to find additional relevant articles. Moreover, references from all related original articles were checked, and previous articles were reviewed to find additional relevant studies.

### 2.2. Inclusion Criteria

Studies were considered eligible if they met the following criteria: (1) All animal models and clinical trials administered garlic in various chemical forms, including raw garlic, fermented garlic, nonfermented garlic, black garlic, diallyl disulfide, and *S*-allyl mercapto cysteine (SAMC); (2) no further treatment should be performed with garlic; and (3) the findings must focus on the effect of garlic on NAFLD and its metabolic comorbidities.

### 2.3. Exclusion Criteria

The exclusion criteria were as follows: (1) Interventions that are not related to garlic, (2) supplements that contain garlic, (3) garlic being used as a remedy for other conditions, (4) presentations, reviews, book chapters, editorials, reports, or commentary papers written in a language other than English, and (5) non-English-language studies.

### 2.4. Data Extraction

An independent review and screening of the full text of the selected studies were carried out by two authors (AK and MV), leading to the extraction of information. Strength of agreement using the kappa coefficient measures the agreement between two reviewers. In this study, kappa statistics were approximately 0.8. The extracted data from the studies included the authors' names, study subjects, dosages, duration, follow-up, and primary outcomes of garlic supplementation. The third author verified the extracted data.

### 2.5. Risk of Bias Assessment

Two independent researchers investigated all the studies for bias (AK and MV). The risk of bias in randomized controlled trials was assessed using the Cochrane risk of the bias assessment method. In contrast, the risk of bias in the included animal studies was assessed using SYRCLE's risk of bias assessment tool. The Cochrane risk of bias (ROB) assessment method was used to assess the overall degree of bias in the randomized controlled trials. Also, the total risk of bias in the animal studies was assessed by SYRCLE's risk of bias tool. In these tools, seven different types of bias were present, including random performance bias, sequence generation bias, attrition bias, detection bias, allocation concealment bias, reporting bias, and other types of bias. There was a “high risk” score for a study when methodological flaws influenced the findings, a “low risk” score for a study without flaws and an “unclear risk” score for a study with insufficient data. Trials with “low risk” in all domains were considered high-quality and low-bias research.

## 3. Results

### 3.1. Literature Search

**Figure 1:**
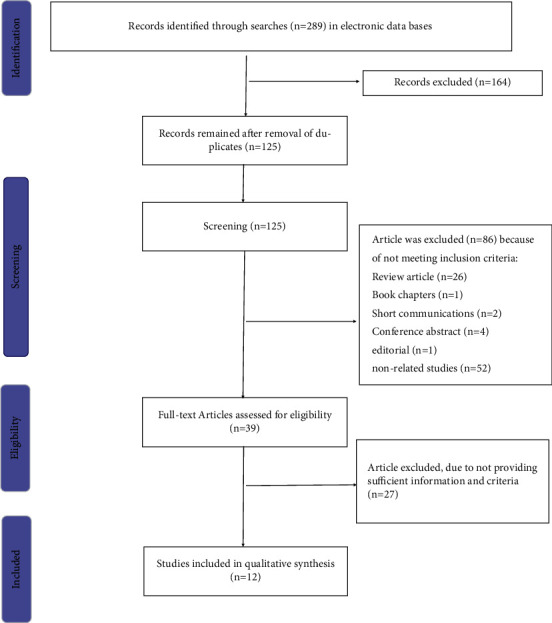
Flowchart of study.

First, two authors independently searched databases for relevant English studies up to the end of February 2022. A total of 289 articles were initially identified from databases (53 from PubMed, 81 from Embase, 85 from Science Direct, 57 from Scopus, and 13 from Cochrane). After eliminating duplicate studies, 125 articles remained to analyze the title and abstract. Finally, 99 studies were considered based on the research topic. After a critical analysis, 12 articles were included in the present study (seven animal and four human studies) (Figure 1).

### 3.2. Findings from the Quality Assessments

**Figure 2:**
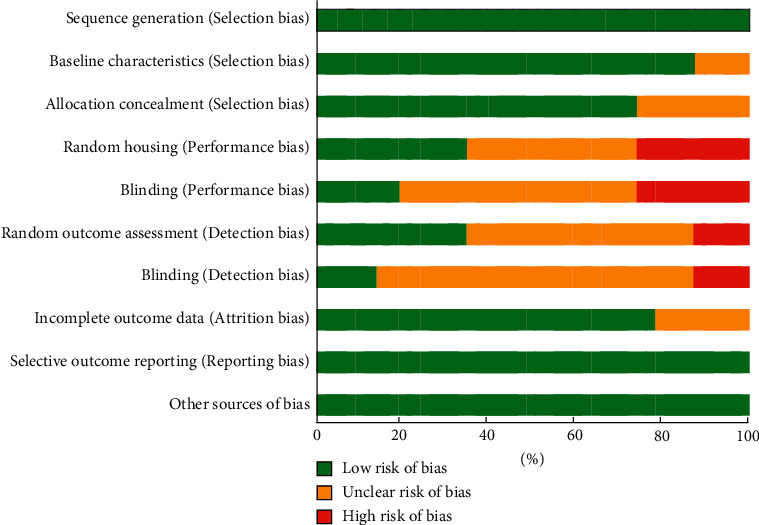
Animal of assess of bias.

**Figure 3:**
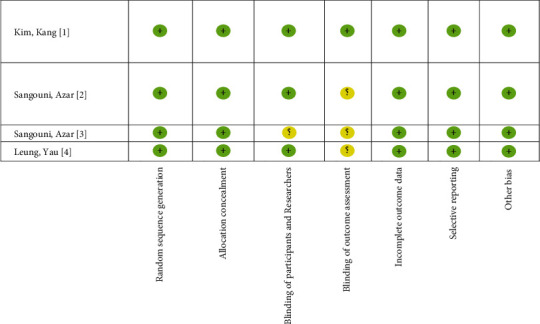
Human assessments of bias.

The results of the methodological quality assessment of included animal and human studies are presented in Figures 2 and 3. In human studies, the results of the blinded outcome assessment showed that three studies were classified as having an unclear risk of bias. The SYRCLE risk of bias tool was used to evaluate the quality of animal studies. The qualitative assessment exhibited that most studies were rated as low risk of bias for the group similarities at baseline category, sequence generation, selective outcome reporting, and other bias category sources. In most of these studies, randomization in animal housing, random outcome assessment, and blinding outcome assessment were not mentioned clearly. Methods of blinding were adequately described in 20% of the included studies. Moreover, the risk of incomplete outcome data was identified in 80% of the studies. Also, the risk of allocation concealment was identified in 70% of the animal studies (Figure 3).

### 3.3. Weight-Lowering Effects of Garlic

There is a direct link between overweight and diabetes. Numerous studies have shown that functional foods and pharmaceutical or dietary supplements effectively treat obesity. There is a direct link between overweight and diabetes. Numerous studies have shown that functional foods and pharmaceutical or dietary supplements effectively treat obesity.

Three human studies investigating the effect of garlic on overweight people with diabetes showed that different doses of garlic (400 mg to 400 g) and the duration of the intervention between 26 days and 12 weeks reduce the body composition and increase the skeletal muscle mass [34–36]. However, in one study, garlic by 100 grams did not affect weight indices for ten weeks [31]. Contrary to the results of the previous study by Fajrani, Sulchan [37] showed that garlic treatment at a dose of 450, 900, and 1350 mg/kg weight for 26 days considerably reduces the body weight of mice.

### 3.4. Garlic and Metabolic Risk Factors Related to NAFLD

#### 3.4.1. Glycemic Parameters

Three animal and two human studies assessed the potential effects of garlic on the glycemic parameters in NAFLD. Studies have evaluated changes in glucose levels, insulin levels, and insulin resistance, as well as the homeostatic model assessment for insulin resistance (HOMA-IR) after the garlic treatment. Animal studies showed that different doses of garlic orally improve the glycemic profile compared to the control group, which may be due to the fact that garlic reduces hepatic glucose secretion and increases glucose uptake by peripheral tissues through increasing insulin signaling and improving blood glucose. They also suggested that garlic might reduce insulin resistance by increasing the peroxisome proliferator-activatedreceptor-gamma (PPAR*γ*) [37–39].

Also, clinical studies showed that garlic in different doses (400 mg to 400 g) and within 12 weeks leads to an improvement in some glycemic parameters [31, 34].

#### 3.4.2. Lipid Profile

Two human and five animal studies assessed lipid profiles following garlic supplementation in NAFLD. Lai et al. [38], Lee et al. [39], Rajaei et al. [36], and Nurmawati et al. [40] showed that different forms of garlic such as *garlic essential oil, black garlic*, and diallyl sulfide lead to a decrease in lipid profile levels and an increase in high-density lipoprotein (HDL). They suggested that garlic may decrease the expression levels of sterol regulatory element-binding protein 1 (SREBP-1c), fatty acid synthase (FAS), acetyl-CoA carboxylase (ACC), and 3-hydroxy-3-methyl-glutaryl and regulate the expression of CoA reductase (HMGCR), leading to an improvement in lipid profile. Kim et al. [31], in a study conducted on NAFLD patients, concluded that fermented garlic extract supplementation for three months did not cause a significant change in lipid profile. In a study conducted on NAFLD patients, Sangouni et al. [35] found that 400 mg/day of garlic powder supplementation for 12 weeks caused a significant decrease in TG, TC, and low-density lipoprotein cholesterol (LDL-C) levels and a significant increase in high-density lipoprotein cholesterol (HDL-C) levels.

#### 3.4.3. Liver Function

Two human and three animal studies assessed lipid profiles following garlic supplementation in NAFLD.

All three animal studies showed that garlic in different doses (200 mg/kg to 500 mg/kg) for eight weeks improves liver enzymes. [39, 41, 42]. Both clinical studies conducted by Kim et al. [31] and Sangouni et al. [35] showed that supplementation with garlic reduces the level of alanine transaminase (ALT) and aspartate transaminase (AST).

### 3.5. Garlic, Inflammation, and Oxidative Stress Indices Related to NAFLD

The effectiveness of garlic on inflammation and oxidative stress in NAFLD was assessed in nine animal studies. Administration of garlic inhibited the expression of inflammation-related genes, including nuclear factor kappa-light-chain (NF-k*β*) and protein kinase B (PKB)/Akt, in NAFLD. Besides, garlic *significantly* decreased serum levels of IL-6, IL-1*β*, tumor necrosis factor (TNF-*α*), and the expression of nuclear factor erythroid 2-related factor 2 (Nrf2).

Xiao et al. [42] found that treatment with garlic for eight weeks significantly decreased the serum levels of inflammatory cytokine, smooth muscle alpha-actin (*α*SMA), malondialdehyde (MDA), cytochrome P450 (CYP2E1), and suppressor of cytokine signaling 3 (SOCS3). Also, it increased the serum levels of adiponectin, glutathione peroxidase (GPx), and CAT in NAFLD rats compared to the control group. Fajrani et al. [37], Yang et al. [43], Lai et al. [38], and Lee et al. [39] reported that supplementation with different doses of garlic resulted in an enhancement in antioxidant enzymes, AMP-activated protein kinase (AMPK), peroxisome proliferator-activated receptor alpha (PPAR-*α*) in NAFLD rats. At the same time, it decreased the oxidative stress indices and some inflammation gene expressions. In addition, in two clinical studies conducted by Kim et al. [31] and Sangouni et al. [34] showed that oral supplementation with 40 g/day garlic for 12 weeks significantly increased the serum levels of TAC and antioxidant enzymes and decreased MDA in NAFLD patients. Moreover, garlic powder significantly increased the expression of TAC and SOD. Other studies are summarized in Tables 1 and 2.

## 4. Discussion

The results of the current systematic review showed that garlic might positively affect NAFLD and its associated metabolic disorders, such as obesity, hyperlipidemia, and insulin resistance. The findings of animal and human studies showed that garlic supplements in different forms, such as black garlic, fermented form, and the active ingredient of garlic, effectively control factors affecting lipid profile and blood sugar. Almost all studies have shown the potential role of garlic in lowering the serum levels of lipids, including TG, LDL-C, TC, and HDL-C. Although in one human study, garlic did not affect the serum levels of TG, LDLLDL, HDL, and TG. Notably, garlic can improve glucose metabolism by reducing insulin resistance, fasting blood glucose, and stimulating glucose uptake by peripheral tissues.

On the other hand, garlic effectively reduces inflammation by affecting the path of NF-*κ*B. However, regarding human studies, only one case has investigated the inflammatory high-sensitivityC-reactive protein (hs-CRP) index. Also, garlic can lead to oxidative balance in the body by inhibiting excessive ROS production. In addition, the results of clinical trials also showed that the MDA changes were insufficient among the included studies. GSTs may improve TAC levels. Therefore, contrary to findings from animal studies, current evidence shows that, in human studies, the effect of garlic supplementation on indicators of oxidative stress, body weight, and inflammation is insufficient. There are inconsistent findings across the included studies regarding antioxidant biomarkers, inflammation, and body weight. This difference can be explained by differences in the dose and duration of interventions, sex, age, genetics, physical activity, nutritional intake, and other confounding factors, such as family history. It is better to conduct studies with the same doses and specific duration. Also, in animal studies, control of physiological conditions and confounding factors are easily controlled compared to human studies. Therefore, it seems that more clinical trial studies are needed.

### 4.1. Possible Mechanisms of Action of Garlic in NAFLD

**Figure 4:**
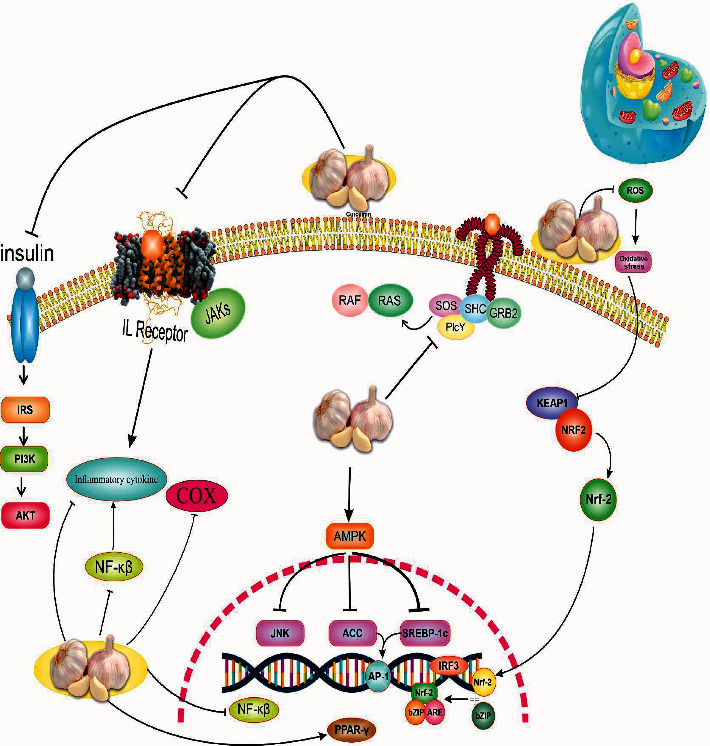
Mechanism of action garlic on a biochemical pathway in NAFLD.

Many studies have shown that plant-based compounds play a therapeutic role in many diseases due to their anti-inflammatory, antioxidative stress, antineurodegenerative, antiaging properties, and improving insulin resistance [45–51]. *Garlic* is a plant that improves lipid profile, blood sugar, inflammation, and oxidative stress [35, 41]. The possible mechanisms of action of garlic in NAFLD are discussed in six major sections, including the effects of garlic on oxidative stress and inflammation, insulin resistance (IR), dyslipidemia, liver enzymes and steatosis, body weight, and modulation of the gut microbiome (Figure 4).

### 4.2. Impacts of Garlic on Oxidative Stress and Inflammation

Out of seven animal studies conducted on oxidative stress and inflammation, all showed that garlic supplementation improves antioxidant status and reduces inflammation. However, in two human studies, garlic reduced oxidative stress and improved antioxidant conditions, which requires more studies to draw better conclusions. Reactive oxygen species (ROS) and inflammation induce oxidative stress, which is the prime mechanism of NAFLD progression and other hepatic diseases [52]. NAFLD is strongly linked to the overproduction of ROS, oxidative stress, and inflammation in the liver [53]. Studies suggested that mitochondrial dysfunction, reduction of intracellular antioxidants, and inflammation are the main reasons behind the excessive production of ROS in NAFLD. Overproduction of ROS also induces lipid peroxidation leading to further cytokine production, fibrogenesis, and hepatocyte apoptosis [52]. Lipid peroxidation establishes a vicious cycle that aggravates mitochondrial dysfunction and oxidative stress [54]. Potential pathways through which garlic can ameliorate oxidative stress have been suggested, including decreasing mitochondrial dysfunction, Kupffer cells activation, gene expression of oxidative stress agents, and increased gene expression of antioxidant agents. Organosulfur compounds (OSCs) are the most effective constituents of garlic that enhance the detoxification system [55]. These compounds stimulate SOD, CAT, and GPx activities [56]. Moreover, *S*-allylcysteine (SAC) attenuates nuclear factor kappa B (NF-кB) activity and, thus, protects cells from ROS-induced injury [57]. Other bioactive compounds, including dietary fibers, microelements (especially selenium), and polyphenols, are also associated with the antioxidant activity of garlic [58]. A meta-analysis study by Moosavian et al. [59] showed that garlic supplementation might improve oxidative stress markers. Also, in another study, Koushki et al. [60] indicated that garlic reduces TNF-*α* and CRP. Consistent with the results obtained from our study, OSCs, such as allicin, diallyl disulfide (DADS), and diallyl trisulfide (DATS), can suppress the production of proinflammatory cytokines and chemokines like IL-1*β*, TNF-*α*, IL-6, IL-10, IL-12, and monocyte chemoattractant protein-1 (MCP-1) [61, 62].

### 4.3. Role of Garlic in Glycemic Indices

In four animal and one human study, supplementation with gallic led to improved glycemic indices and insulin resistance. NAFLD is tightly related to IR and decreased insulin sensitivity at both the muscle level and the hepatic and adipose tissue levels [63]. Hyperglycemia and hyperinsulinemia that occur as a result of IR can promote hepatic steatosis. In addition, it can contribute to higher ROS levels due to the reduction in mitochondrial beta-oxidation and activation of other oxidation pathways [37, 64]. Excessive supply of free fatty acids (FFAs) to the liver, increased release of reactive oxygen metabolites (ROMs), and changes in secretion and action of adipocytokines are involved in the pathogenesis of insulin signaling defect in NAFLD [65]. A possible mechanism regarding the effect of garlic on IR is that garlic can decrease the activation of enzymes involved in the formation of fat in the liver and regulate lipogenesis [34]. In addition, adiponectin, as an insulin sensitizer, functions by activating the AMPK and the phosphorylation of ACC in the liver and skeletal muscle [11, 13]. The circulatory levels of adiponectin decrease in NAFLD [64, 65]. Since garlic can lead to higher adiponectin concentration, it can improve IR [34]. SAC can also impose an insulin-like impact on peripheral tissues by promoting glucose uptake and metabolism or preventing hepatic gluconeogenesis [66].

Moreover, garlic oil and DATS have an additional favorable effect on insulin secretion and sensitivity via their antioxidant capacity [67]. In a study conducted on diabetic rats, garlic oil and DATS did not significantly impact fasting blood glucose [67]. Nevertheless, in a study on rats with NAFLD, SAC could improve HbA1c and blood glucose levels [68]. These contradictory findings are partly attributed to different ways of garlic preparation and route of intervention, dose, and duration of treatment [67].

### 4.4. Effect of Garlic on Dyslipidemia

Of the seven studies conducted to investigate the effect of garlic on lipid profiles and dyslipidemia, all studies showed the influential role of garlic in blood lipid indicators. However, one human study improved lipid profile, and another human study could not find a significant effect on the lipid profile. Dyslipidemia in NAFLD is defined as high levels of TG, LDL-C, small-density LDL (sdLDL) particles, and decreased concentrations of high-density lipoprotein cholesterol (HDL-C) [69]. This atherogenic dyslipidemia is linked to CVD risk factors in NAFLD patients [69]. IR promotes an increase in FFA flux, which stimulates TG and very-low-density lipoprotein (VLDL) production and triggers oxidative stress and lipid peroxidation [70]. Hepatic overproduction of VLDL and disrupted clearance of triglyceride-rich lipoproteins from the circulation are the underlying reasons for dyslipidemia in NAFLD [71, 72]. Several mechanisms have been proposed for the antihyperlipidemic effects of garlic. Garlic reduces the expression of the intestinal microsomal triglyceride transfer protein (MTP) gene and suppresses the aggregation and secretion of chylomicrons from the intestine to the blood circulation [73]. Another mechanism is that OSCs exhibit lipid-lowering effects via the inhibition of 3-hydroxy-3-methyl-glutaryl-CoA reductase (HMGR) activity by decreasing the levels of HMGR mRNA, which is mediated by the inactivation of sterol regulatory element-bindingprotein-2 (SREBP-2) and by preventing the activation of cAMP response element-binding protein (CREB) [74].

Furthermore, garlic may exert a lipid-lowering impact by increasing adiponectin levels [34]. Adiponectin activates AMPK and PPAR-*α*, inducing fatty acid oxidation in the liver and muscle. Adiponectin concentrations also have an inverse association with plasma TGs and positively correlate with HDL-C and LDL-C sizes [63]. Regarding the effect of garlic on lipid profile, there are some discrepancies among several publications. Soleimani et al. [75] demonstrated that garlic administration significantly reduces TC, LDL-C, and TG concentrations in NAFLD patients, while contrary to Soleimani et al.'s [75] study, the results of Jung et al. [76] reported no significant improvement in TG, LDL-C, and TC in patients with mild hypercholesterolemia. In a review study, Sobenin et al. [77]showed that garlic could improve the lipid profile. However, in a meta-analysis study, Khoo et al. [78] showed that garlic does not affect lipid profile. These different results might be due to different types of garlic, different study designs, baseline lipid profiles, and study populations [34, 75].

### 4.5. Impacts of Garlic on Liver Enzymes and Steatosis

Four animal studies and two human studies conducted to investigate the effect of garlic on liver enzymes showed that garlic could play an influential role in controlling and improving liver enzymes and steatosis. Hepatic steatosis, the accumulation of TG in hepatocytes, is a major reason for increased liver enzymes [79, 80]. However, it is noteworthy to mention that up to one-third of NAFLD patients do not have any enzyme abnormalities even with the development of NASH [81]. When the balance between four pathways, namely, the uptake of circulating lipids, de novo lipogenesis, fatty acid oxidation, and export of lipids to VLDL, is dysregulated, hepatic steatosis occurs [54]. Garlic regulates lipogenesis by reducing the activity of enzymes in hepatic fat production, IR, NFкB pathway, and gene expression of oxidative stress markers and contributes to hepatic steatosis improvement [34]. Therefore, garlic affects hepatic steatosis and liver enzymes through multiple pathways. For instance, allicin displays antioxidant and anti-inflammatory effects by downregulating the expression of NFкB and suppressing the Jun N-terminal kinase (JNK) pathway [82, 83]. Additionally, downregulation of lipogenic gene expressions, such as fatty acid synthase and ACC via SREBP1c, and upregulation of lipolytic gene expressions, like PPAR-*α* and carnitine palmitoyltransferase-1(CPT-1), are other functions of garlic [38, 84]. Ajoene, a stable OSC, may also upregulate the expression of nuclear factor erythroid-2-related factor 2 (Nrf2), which is involved in synthesizing detoxification enzymes and glutathione [85].

### 4.6. Garlic and Body Weight

Two of the eight animal studies that examined garlic's effect on body weight found that garlic had no significant effect on weight. However, unlike animal studies, in two out of three human studies that investigated the effect of garlic on anthropometric indices, the results showed that garlic could have a positive effect on improving anthropometric indices. One of the most significant factors associated with NAFLD pathogenesis is obesity [34]. Several studies have reported the antiobesity effects of garlic [34, 38]. One of the explanations for the antiobesity property of garlic is its thermogenic trait, which can elevate energy expenditure by upregulating the expression of the uncoupling protein-2 (UCP-2) gene [79, 86]. Besides, inhibiting adipogenesis and adipocyte differentiation through downregulation of PPAR*γ*, SREBP-1c, and fatty acid-binding protein (aP2) are other beneficial effects of garlic in obesity treatment [37, 79]. Khoo and Aziz [78] indicated that garlic supplementation might reduce waist circumference without affecting body weight and BMI. These different results may be due to different types of garlic, different duration of the study, and amount of dosage.

### 4.7. Garlic and Modulation of the Gut Microbiome

Increasing evidence suggests that dysbiosis is linked to NAFLD and its severity [87–89]. Alterations of gut microbiota increase intestinal permeability and the translocation of lipopolysaccharides (LPS) derived from Gram-negative bacteria surfaces into the blood. This causes endotoxemia, leading to hepatic inflammation [90, 91]. Furthermore, dysbiosis might give rise to intestinal short-chain fatty acid (SCFA) and bile acid profile changes. Because increased bile acid production can stimulate the epidermal growth factor receptor (EGFR), impaired gut permeability and NASH will ensue [91, 92]. The prebiotic effect of garlic can increase microbial richness and diversity, particularly by stimulating the growth of *Lactobacillus* and *Clostridia* species [93]. Although the study by Yang et al. [43] showed that a low dose of DADS induced fatty liver and gut microbiota alteration, Chen et al. [94] reported that whole garlic supplementation increased gut microbiome diversity, especially f_*Lachnospiraceae,* and decreased the frequency of g_*Prevotella*. This inconsistency indicates that fructan and OSCs derived from garlic have opposite effects on the frequency of f_*Lachnospiraceae*. Therefore, the whole garlic intake can elevate the frequency of f_*Lachnospiraceae* [94].

### 4.8. Strengths, Limitations, Future Directions, and Knowledge Gaps

The present study has both limitations and strengths. The study reviewed both animal and human studies with sufficient sample sizes. Despite its strengths, the study's main limitation was the heterogeneity of selected studies, which could be due to factors including variation in doses and study durations. Due to the administration of various dosages and models of garlic in experimental studies, the lack of information regarding the required dosage ranges is considerable, which is needed to be elucidated. The current review included all the eligible animal and human studies assessing the effect of garlic on NAFLD by providing the majority of potential mechanisms of action of garlic in various NAFLD outcomes. However, due to different administered compounds and doses and duration of intervention, it might be difficult to compare the results of included publications. Therefore, future clinical trials with larger sample sizes and extended intervention periods are warranted (Table 2).

## 5. Conclusion

In conclusion, garlic used in the reviewed papers had various beneficial effects on NAFLD by mitigating oxidative stress, IR, dyslipidemia, hepatic injury, and gut dysbiosis. No adverse side effects were reported.

## Figures and Tables

**Table 1:** Characteristics of studies investigating the potential role of garlic in NAFLD.

Author's name	Study design	Number and type of subjects	Dosage and type of administration	Assay	Study duration	Route	Main results
Animal studies							
Xiao et al. [42]	In vivo	Rats (*n* = 7)	200 mg/kg SAMC	Transmission electron microscopy, RNA isolation and analysis, real-time PCR, ELISA, histopathological examination, protein determination, western blot	8 weeks	Oral	(i) Decrease in the concentrations/activities/expressions ofSREBP1c, TNF-*α*, IL-1b, iNOS, COX-2.MCP-1, MIP-2, IL-6, TGF-b, PC-1, ALT, FFAS, *α*SMA, MDA, CYP2E1, NTR formation, SOCS3 protein adducts in the live. (ii) An increase in adiponectin, GPx, and CAT concentrations

Lee et al. [39]	In vivo	Mice (*n* = 6)	500 mg/kg	ELISA, western blot, real-time PCR, histological analysis, ovarian morphological analysis, measurement of hormones, measurement of the ROS level and mitochondrial membrane potential, plasmid construction and luciferase assays	8 weeks	Oral	(i) Reduction of the elevated serum AST, ALT, and GGT activities as well as TG, TC, serum glucose, serum insulin, and HOMA-IR, concentrations also decrease liver steatosis, liver weight ,(ii) Reduction in the elevated ACC, CPT-1 JNK, pJNK, TNF-*α*, SREBP-1c, SCD-1, MDA, BAX, caspase 3 and enhancement of the liver glutathione content, AMPK, PPAR-*α*

Fajrani et al. [37]	In vivo	Rats (*n* = 18)	450, 900, and 1350 mg/kg	ELISA, histological analysis	26 days	Oral	(i) Decrease in serum concentrations of MDA, HOMA-IR, insulin, and FBG and decrease in body weight and visceral fat

Yang et al. [43]	In vivo	Rats (*n* = 12)	10 and 20 mg/kg	ELISA, gene expression, histological analysis, assay of malondialdehyde and protein carbonyl, oxidative stress measurement	8 weeks	Oral	(ii) Decrease in serum concentrations of LDL-C, TG, TC, FBG, PPAR*γ* hepatic malondialdehyde content, and increase in HDL-C; (iii) The effect of regulation of lipid metabolism via a connection with the regulation of ACC *α*1, ACC *β*1, FASN, DGAT, SREBP-1 and 2, HMG-CoAR, SCD 1, and 1 in hepatic tissue

Lai et al. [38]	In vivo	Rats (*n* = 30)	25, 50, and 100 mg/kg	ELISA, assay of glutathione, malondialdehyde, protein carbonyl, and oxidative stress measurement	12 weeks	Oral	(i) A reduction in the expression of genes related to fatty acid (SREBP-1c, FAS, ACC, HMGCR, CPT-1, and CYP2E1) pathways, reducing the composition of plasma fatty acids (LDL-C, TG, TC) and the expression of relevant genes including TNF-*α*, IL-1*β*, and IL-6, and inhibition of oxidative stress-related biomarker concentrations (ii) Decrease in serum concentrations of LDL-C, TG, TC, FBG, PPAR*γ* and increase in HDL-C, antioxidative enzymes, namely, GSH, SOD, CAT, catalase, GPx, GRd, GST, PPAR
Nurmawati et al. [40]	In vivo	Rats (*n* = 18)	450, 900 and 1350 mg/kg	ELISA	4 weeks	Oral	(i) Decrease in serum concentrations of PAI-1 TC, LDL-C TG and increase in HDL-C level

Seif El-Din et al. [41]	In vivo	Rats (*n* = 10)	500 mg/kg/day	ELISA, western blot, real-time PCR	8 weeks	Oral	(i) Decrease in activities of serum ALT, AST, ALP, leptin, cholesterol, triglycerides, TNF-*α*, TGF-*β*, and the concentrations of hepatic MDA. (ii) Increase in the activities of GR, GST, SOD, and glutathione peroxidase as well as the concentrations of glutathione (iii) There was no change in body weight and ovaries.

Wu et al. [44]	In vivo	Rats (*n* = 10)	100 mg/kg b.w./d i.p.	ELISA, western blot, real-time PCR	8 weeks	Oral	(i) A decrease in the levels/activities of ALT, AST, MDA, COX-2 (ii) Elevation in the activities of SOD

Akt: protein kinase B, AMH: anti-mullerian hormone, AMPK: AMP-activated protein kinase, BAX: BCL-2-associated X-protein, BMI: body mass index, CYP11A1: cytochrome P450 family 11 subfamily A member 1, CYP17A1: cytochrome P450 17A1, CYP21: cytochrome P450c21, CAT: catalase, COX: cyclooxygenase, ELISA: enzyme-linked immunosorbent assay, FBG: fasting blood glucose, FSH: follicle-stimulating hormone, GKG: glucokinase, GPX: glutathione peroxidase, HDL-C: high-density lipoprotein cholesterol, HMGCR: hydroxy-3-methylglutaryl-CoA, HOMA-IR: homeostasis model of assessment-insulin resistance, HSD3B1: hydroxy-delta-5-steroid dehydrogenase, 3 beta- and steroid delta-isomerase 1, ICP-OES: inductively coupled plasma optical emission spectrometry, IGF1: insulin like growth factor 1, IL: interleukin, IR: insulin resistance, LDL-C: low-density lipoprotein cholesterol, LH: luteinizing hormone, MDA: malondialdehyde, NF-ΚB: nuclear factor-*κB*, PCR: polymerase chain reaction, PI3K: phosphatidylinositol 3-kinase, RNS: reactive nitrogen species, ROS: reactive oxygen species, SIRT: sirtuin, SOD: super oxide dismutase, TAC: total antioxidant capacity, TGF-*β*: transforming growth factor *β*, TNF-*α*: tumor necrosis factor *α*, TG: triglycerides, TC: total cholesterol, VLDL: very-low-density lipoprotein, VEGF: vascular endothelial growth factor, and VEGF: vascular endothelial growth factor.

**Table 2:** Summary of the human studies on the effect of garlic on NAFLD.

Reference	Number and type of subjects	Sample size	Age	Dosage and type of administration	Sources	Study duration	Maine outcome
Kim et al. [31]	Human with NAFLD (*n* = 36)	75	20–75	40 g/day	China	12 weeks	Significant decrease: GGT, ALT significant increase Insulin sensitivity, GSH, TAC, shbg, Nonsignificant change: hs-CRP, LDL, HDL, TG, DHEAS, BMI, WC, HC

Sangouni et al. [35]	Human with NAFLD (*n* = 45)	88	≥18	400 mg/day	Iran	12 weeks	Significant decrease: GGT, ALT, AST, TG, TC, LDL, liver steatosis, weight; significant increase: HDL Nonsignificant change: ALP

Sangouni et al. [34]	Human with NAFLD (*n* = 45)	88	≥18	400 mg/day	Iran	12 weeks	Significant increase: TAC, SOD, skeletal muscle mass Significant decrease: HOMA-IR, insulin, FBS, MDA, waist circumference, body fat percent

Leung et al. [95]	Human with NAFLD (*n* = 41)	41	≥18	1080 mg	Iran	13 weeks	Significant increase: *α*-linolenic acid arachidic acid Significant decrease: Palmitoleic acid, 27-hydroxycholesterol, 7*β*-hydroxycholesterol, 7-ketocholesterol, 5-HETE and 12-HETE, 9-HETE, and 11-HETE

Family 11 subfamily A member 1, CYP17A1: cytochrome P450 17A1, CYP21: cytochrome P450c21, CAT: catalase, COX: cyclooxygenase, assay, FBG: fasting blood glucose, FSH: follicle-stimulating hormone, GKG: glucokinase, GPX: glutathione peroxidase, HDL-C: high-density lipoprotein cholesterol, HMGCR: hydroxy-3-methylglutaryl-CoA, HOMA-IR: homeostasis model of assessment-insulin resistance, HSD3B1: hydroxy-delta-5-steroid dehydrogenase, 3 beta- and steroid delta-isomerase 1, ICP-OES: inductively coupled plasma optical emission spectrometry, IGF1: insulin like growth factor 1, IL: interleukin, IR: insulin resistance, LDL-C: low-density lipoprotein cholesterol, LH: luteinizing hormone, and MDA: malondialdehyde.

## Data Availability

All data generated or analyzed during this study are included within the article.

## Supplementary Materials

Supplementary Materials
*Supplementary Table 1.* Search strategies including the key terms and the queries for each database.

## References

[1] Tretyakova V. A., Zhernakova N. I., Arisheva O. S. (2022). Meta-analysis of non-alcoholic fatty liver disease and electromechanical reconstruction of myocardium. *Archives of Razi Institute*.

[2] Chalasani N., Younossi Z., Lavine J. E. (2012). The diagnosis and management of non‐alcoholic fatty liver disease: practice guideline by the American association for the study of liver diseases, American college of gastroenterology, and the American gastroenterological association. *Hepatology*.

[3] Williams C. D., Stengel J., Asike M. I. (2011). Prevalence of nonalcoholic fatty liver disease and nonalcoholic steatohepatitis among a largely middle-aged population utilizing ultrasound and liver biopsy: a prospective study. *Gastroenterology*.

[4] Benedict M., Zhang X. (2017). Non-alcoholic fatty liver disease: an expanded review. *World Journal of Hepatology*.

[5] Peverill W., Powell L. W., Skoien R. (2014). Evolving concepts in the pathogenesis of NASH: beyond steatosis and inflammation. *International Journal of Molecular Sciences*.

[6] Nomura K., Yamanouchi T. (2012). The role of fructose-enriched diets in mechanisms of nonalcoholic fatty liver disease. *Journal of Nutritional Biochemistry*.

[7] Kooshki F., Tutunchi H., Vajdi M. (2021). A Comprehensive insight into the effect of chromium supplementation on oxidative stress indices in diabetes mellitus: a systematic review. *Clinical and Experimental Pharmacology and Physiology*.

[8] Harmon R. C., Tiniakos D. G., Argo C. K. (2011). Inflammation in nonalcoholic steatohepatitis. *Expert Review of Gastroenterology & Hepatology*.

[9] Kooshki F., Moradi F., Karimi A. (2020). Chromium picolinate balances the metabolic and clinical markers in nonalcoholic fatty liver disease: a randomized, double-blind, placebo-controlled trial. *European Journal of Gastroenterology and Hepatology*.

[10] Tilg H., Moschen A. R. (2010). Evolution of inflammation in nonalcoholic fatty liver disease: the multiple parallel hits hypothesis. *Hepatology*.

[11] Vos B., Moreno C., Nagy N. (2011). Lean non-alcoholic fatty liver disease (Lean-NAFLD): a major cause of cryptogenic liver disease. *Acta Gastroenterol Belg*.

[12] Lakka H.-M., Laaksonen D. E., Lakka T. A. (2002). The metabolic syndrome and total and cardiovascular disease mortality in middle-aged men. *JAMA*.

[13] Oseini A. M., Sanyal A. J. (2017). Therapies in non-alcoholic steatohepatitis (NASH). *Liver International*.

[14] Rahman M. M., Ferdous K. S., Ahmed M. (2020). Emerging promise of nanoparticle-based treatment for Parkinson’s disease. *Biointerface Research in Applied Chemistry*.

[15] Rahman M. M., Islam F., Parvez A. (2022). Citrus limon L. (lemon) seed extract shows neuro-modulatory activity in an in vivo thiopental-sodium sleep model by reducing the sleep onset and enhancing the sleep duration. *Journal of Integrative Neuroscience*.

[16] Islam F., Nafady M. H., Islam M. R. (2022). Resveratrol and neuroprotection: an insight into prospective therapeutic approaches against Alzheimer’s disease from bench to bedside. *Molecular Neurobiology*.

[17] Karimi A., Tutunchi H., Naeini F., Vajdi M., Mobasseri M., Najafipour F. (2022). The therapeutic effects and mechanisms of action of resveratrol on polycystic ovary syndrome: a comprehensive systematic review of clinical, animal and in vitro studies. *Clinical and Experimental Pharmacology and Physiology*.

[18] Karimi A., Niazkar H. R., Sefidmooye Azar P. (2021). Protective effect of hydro-alcoholic extract of *achillea millefolium* on renal injury and biochemical factors in streptozotocin-induced diabetic rats. *Nutrition & Food Science*.

[19] Kooshki F., Niazkar H. R., Shirazi S., Asghari Azar V., Karimi A. (2020). Fumaria parviflora improves liver damage and lipid profile changes in STZ-induced diabetic rats. *Physiology and Pharmacology*.

[20] Nasimi Doost Azgomi R., Karimi A., Tutunchi H., Moini Jazani A. (2021). A comprehensive mechanistic and therapeutic insight into the effect of chicory (Cichorium intybus) supplementation in diabetes mellitus: a systematic review of literature. *International Journal of Clinical Practice*.

[21] Tajaddini A., Roshanravan N., Mobasseri M. (2021). Saffron improves life and sleep quality, glycaemic status, lipid profile and liver function in diabetic patients: a double-blind, placebo-controlled, randomised clinical trial. *International Journal of Clinical Practice*.

[22] Cicero A. F., Colletti A., Bellentani S. (2018). Nutraceutical approach to non-alcoholic fatty liver disease (NAFLD): the available clinical evidence. *Nutrients*.

[23] Valenti L. (2013). *Dietary anthocyanins as nutritional therapy for nonalcoholic fatty liver disease*.

[24] Abenavoli L., Milic N., Luzza F., Boccuto L., De Lorenzo A. (2017). Polyphenols treatment in patients with nonalcoholic fatty liver disease. *Journal of translational internal medicine*.

[25] Bayan L., Koulivand P. H., Gorji A. (2014). Garlic: a review of potential therapeutic effects. *Avicenna journal of phytomedicine*.

[26] Iciek M., Kwiecień I., Włodek L. (2009). Biological properties of garlic and garlic‐derived organosulfur compounds. *Environmental and Molecular Mutagenesis*.

[27] El-Bayoumy K., Sinha R., Pinto J. T., Rivlin R. S. (2006). Cancer chemoprevention by garlic and garlic-containing sulfur and selenium compounds. *Journal of Nutrition*.

[28] Tripathi P., Gupta P. P., Lal V. K. (2013). Effect of Co-administration of Allium sativum extract and Metformin on Blood glucose of Streptozotocin induced diabetic rats. *Journal of Intercultural Ethnopharmacology*.

[29] Chan J. Y. Y., Yuen A. C. Y., Chan R. Y. K., Chan S. W. (2013). A review of the cardiovascular benefits and antioxidant properties of allicin. *Phytotherapy Research*.

[30] Maeda T., Miki S., Morihara N., Kagawa Y. (2019). Aged garlic extract ameliorates fatty liver and insulin resistance and improves the gut microbiota profile in a mouse model of insulin resistance. *Experimental and Therapeutic Medicine*.

[31] Kim H.-N., Kang S. G., Roh Y. K., Choi M. K., Song S. W. (2017). Efficacy and safety of fermented garlic extract on hepatic function in adults with elevated serum gamma-glutamyl transpeptidase levels: a double-blind, randomized, placebo-controlled trial. *European Journal of Nutrition*.

[32] Padiya R., Khatua T. N., Bagul P. K., Kuncha M., Banerjee S. K. (2011). Garlic improves insulin sensitivity and associated metabolic syndromes in fructose fed rats. *Nutrition & Metabolism*.

[33] Paknahad Z., Soleimani D., Askari G., Iraj B., Feizi A. (2016). Effect of garlic powder consumption on body composition in patients with nonalcoholic fatty liver disease: a randomized, double-blind, placebo-controlled trial. *Advanced Biomedical Research*.

[34] Sangouni A. A., Mohammad Hosseini Azar M. R., Alizadeh M. (2020). Effects of garlic powder supplementation on insulin resistance, oxidative stress, and body composition in patients with non-alcoholic fatty liver disease: a randomized controlled clinical trial. *Complementary Therapies in Medicine*.

[35] Sangouni A. A., Mohammad Hosseini Azar M. R., Alizadeh M. (2020). Effect of garlic powder supplementation on hepatic steatosis, liver enzymes and lipid profile in patients with non-alcoholic fatty liver disease: a double-blind randomised controlled clinical trial. *British Journal of Nutrition*.

[36] Rajaei S., Alihemmati A., Abedelahi A. (2019). Antioxidant effect of genistein on ovarian tissue morphology, oxidant and antioxidant activity in rats with induced polycystic ovary syndrome. *International Journal of Reproductive BioMedicine*.

[37] Fajrani A. M., Sulchan M., Muis S. F. (2021). Effect of black garlic on visceral fat, oxidative stress and insulin resistance in nonalcoholic fatty liver disease rats. *Nutrition & Food Science*.

[38] Lai Y.-S., Chen W. C., Ho C. T. (2014). Garlic essential oil protects against obesity-triggered nonalcoholic fatty liver disease through modulation of lipid metabolism and oxidative stress. *Journal of Agricultural and Food Chemistry*.

[39] Lee H.-S., Lee S. J., Yu H. J., Lee J. H., Cho H. Y. (2017). Fermentation with Lactobacillus enhances the preventive effect of garlic extract on high fat diet-induced hepatic steatosis in mice. *Journal of Functional Foods*.

[40] Nurmawati L., Sulchan M., Fatimah-Muis S. (2021). The effect of single clove black garlic on the hemostasis status and lipid profile in male sprague dawley rats with NON-alcoholic fatty liver disease. *Potravinarstvo Slovak Journal of Food Sciences*.

[41] Seif El-Din S. H., Sabra A. N. A., Hammam O. A., Ebeid F. A., El-Lakkany N. M. (2014). Pharmacological and antioxidant actions of garlic and/or onion in non-alcoholic fatty liver disease (NAFLD) in rats. *Journal of the Egyptian Society of Parasitology*.

[42] Xiao J., Ching Y. P., Liong E. C., Nanji A. A., Fung M. L., Tipoe G. L. (2013). Garlic-derivedS-allylmercaptocysteine is a hepato-protective agent in non-alcoholic fatty liver disease in vivo animal model. *European Journal of Nutrition*.

[43] Yang Y., Yang F., Huang M. (2019). Fatty liver and alteration of the gut microbiome induced by diallyl disulfide. *International Journal of Molecular Medicine*.

[44] Wu Z. R., Chen P., Yang L. (2015). Two cinnamoyloctopamine antioxidants from garlic skin attenuates oxidative stress and liver pathology in rats with non-alcoholic steatohepatitis. *Phytomedicine*.

[45] Rahman M. M., Islam M. R., Shohag S. (2022). Microbiome in cancer: role in carcinogenesis and impact in therapeutic strategies. *Biomedicine & Pharmacotherapy*.

[46] Rahman M. M., Bibi S., Rahaman M. S. (2022). Natural therapeutics and nutraceuticals for lung diseases: traditional significance, phytochemistry, and pharmacology. *Biomedicine & Pharmacotherapy*.

[47] Rahman M. M., Dhar P. S., Sumaia (2022). Exploring the plant-derived bioactive substances as antidiabetic agent: an extensive review. *Biomedicine & Pharmacotherapy*.

[48] Mominur Rahman M., Islam F., Saidur Rahaman M., Sultana N. A., Fahim N. F., Ahmed M. (2021). Studies on the prevalence of HIV/AIDS in Bangladesh including other developing countries. *Advances in Traditional Medicine*.

[49] Islam F., Mitra S., Nafady M. H. (2022). Neuropharmacological and antidiabetic potential of lannea coromandelica (houtt.) merr. Leaves extract: an experimental analysis. *Evidence-based Complementary and Alternative Medicine*.

[50] Islam F., Khadija J. F., Harun-Or-Rashid M. (2022). Bioactive compounds and their derivatives: an insight into prospective phytotherapeutic approach against alzheimer’s disease. *Oxidative Medicine and Cellular Longevity*.

[51] Akter A., Islam F., Bepary S. (2022). CNS depressant activities of Averrhoa carambola leaves extract in thiopental-sodium model of Swiss albino mice: implication for neuro-modulatory properties. *Biologia*.

[52] Farzanegi P., Dana A., Ebrahimpoor Z., Asadi M., Azarbayjani M. A. (2019). Mechanisms of beneficial effects of exercise training on non-alcoholic fatty liver disease (NAFLD): roles of oxidative stress and inflammation. *European Journal of Sport Science*.

[53] Braud L., Battault S., Meyer G. (2017). Antioxidant properties of tea blunt ROS-dependent lipogenesis: beneficial effect on hepatic steatosis in a high fat-high sucrose diet NAFLD obese rat model. *The Journal of Nutritional Biochemistry*.

[54] Ipsen D. H., Lykkesfeldt J., Tveden-Nyborg P. (2018). Molecular mechanisms of hepatic lipid accumulation in non-alcoholic fatty liver disease. *Cellular and Molecular Life Sciences*.

[55] Oktari K. (2020). A Review: Antioxidant and Immunomodulator Effects of Black Garlic. https://www.google.com/search?q=A+Review%3A+Antioxidant+and+Immunomodulator+Effects+of+Black+Garlic&rlz=1C1GCEB_enIN993IN993&oq=A+Review%3A+Antioxidant+and+Immunomodulator+Effects+of+Black+Garlic&aqs=chrome.0.69i59.20072j0j9&sourceid=chrome&ie=UTF-8.

[56] Trio P. Z., You S., He X., He J., Sakao K., Hou D. X. (2014). Chemopreventive functions and molecular mechanisms of garlic organosulfur compounds. *Food & Function*.

[57] Geng Z., Rong Y., Lau B. H. (1997). S-allyl cysteine inhibits activation of nuclear factor kappa B in human T cells. *Free Radical Biology and Medicine*.

[58] Lanzotti V. (2006). The analysis of onion and garlic. *Journal of Chromatography A*.

[59] Moosavian S. P., Arab A., Paknahad Z., Moradi S. (2020). The effects of garlic supplementation on oxidative stress markers: a systematic review and meta-analysis of randomized controlled trials. *Complementary Therapies in Medicine*.

[60] Koushki M., Amiri-Dashatan N., Pourfarjam Y., Doustimotlagh A. H. (2021). Effect of garlic intake on inflammatory mediators: a systematic review and meta-analysis of randomised controlled trials. *Postgraduate Medical Journal*.

[61] Lang A. (2004). Allicin inhibits spontaneous and TNF-*α* induced secretion of proinflammatory cytokines and chemokines from intestinal epithelial cells. *Clinical Nutrition*.

[62] You S., Nakanishi E., Kuwata H. (2013). Inhibitory effects and molecular mechanisms of garlic organosulfur compounds on the production of inflammatory mediators. *Molecular Nutrition & Food Research*.

[63] Gaggini M., Morelli M., Buzzigoli E., DeFronzo R., Bugianesi E., Gastaldelli A. (2013). Non-alcoholic fatty liver disease (NAFLD) and its connection with insulin resistance, dyslipidemia, atherosclerosis and coronary heart disease. *Nutrients*.

[64] Milić S., Lulić D., Štimac D. (2014). Non-alcoholic fatty liver disease and obesity: biochemical, metabolic and clinical presentations. *World Journal of Gastroenterology*.

[65] Polyzos S. A., Kountouras J., Zavos C. (2009). Nonalcoholic fatty liver disease: the pathogenetic roles of insulin resistance and adipocytokines. *Current Molecular Medicine*.

[66] Saravanan G., Ponmurugan P. (2011). Ameliorative potential of S-allyl cysteine on oxidative stress in STZ induced diabetic rats. *Chemico-Biological Interactions*.

[67] Liu C.-T., Hse H., Lii C. K., Chen P. S., Sheen L. Y. (2005). Effects of garlic oil and diallyl trisulfide on glycemic control in diabetic rats. *European Journal of Pharmacology*.

[68] Takemura S., Minamiyama Y., Kodai S. (2013). S-Allyl cysteine improves nonalcoholic fatty liver disease in type 2 diabetes Otsuka Long-Evans Tokushima Fatty rats via regulation of hepatic lipogenesis and glucose metabolism. *Journal of Clinical Biochemistry & Nutrition*.

[69] Katsiki N., Mikhailidis D. P., Mantzoros C. S. (2016). Non-alcoholic fatty liver disease and dyslipidemia: an update. *Metabolism*.

[70] Zhang Q. Q., Lu L.-G. (2015). Nonalcoholic fatty liver disease: dyslipidemia, risk for cardiovascular complications, and treatment strategy. *Journal of Clinical and Translational Hepatology*.

[71] Taskinen M.-R., Adiels M., Westerbacka J. (2011). Dual metabolic defects are required to produce hypertriglyceridemia in obese subjects. *Arteriosclerosis, Thrombosis, and Vascular Biology*.

[72] Chatrath H., Vuppalanchi R., Chalasani N. (2012). Dyslipidemia in patients with nonalcoholic fatty liver disease. *Seminars in Liver Disease*.

[73] Lin M. C., Wang E. J., Lee C. (2002). Garlic inhibits microsomal triglyceride transfer protein gene expression in human liver and intestinal cell lines and in rat intestine. *Journal of Nutrition*.

[74] Rai S. K., Sharma M., Tiwari M. (2009). Inhibitory effect of novel diallyldisulfide analogs on HMG-CoA reductase expression in hypercholesterolemic rats: CREB as a potential upstream target. *Life Sciences*.

[75] Soleimani D., Paknahad Z., Rouhani M. H. (2020). Therapeutic effects of garlic on hepatic steatosis in nonalcoholic fatty liver disease patients: a randomized clinical Trial. *Diabetes, Metabolic Syndrome and Obesity: Targets and Therapy*.

[76] Jung E.-S., Park S. H., Choi E. K. (2014). Reduction of blood lipid parameters by a 12-wk supplementation of aged black garlic: a randomized controlled trial. *Nutrition*.

[77] Sobenin I. A., Myasoedova V. A., Iltchuk M. I., Zhang D. W., Orekhov A. N. (2019). Therapeutic effects of garlic in cardiovascular atherosclerotic disease. *Chinese Journal of Natural Medicines*.

[78] Khoo Y. S. K., Aziz Z. (2009). Garlic supplementation and serum cholesterol: a meta-analysis. *Journal of Clinical Pharmacy and Therapeutics*.

[79] Lee M.-S., Kim I. H., Kim C. T., Kim Y. (2011). Reduction of body weight by dietary garlic is associated with an increase in uncoupling protein mRNA expression and activation of AMP-activated protein kinase in diet-induced obese mice. *Journal of Nutrition*.

[80] Markus M. R. P., Meffert P. J., Baumeister S. E. (2016). Association between hepatic steatosis and serum liver enzyme levels with atrial fibrillation in the general population: the Study of Health in Pomerania (SHIP). *Atherosclerosis*.

[81] Wang X. J., Malhi H. (2018). Nonalcoholic fatty liver disease. *Annals of Internal Medicine*.

[82] Rahman M. S. (2007). Allicin and other functional active components in garlic: health benefits and bioavailability. *International Journal of Food Properties*.

[83] Li C. (2015). Allicin Alleviates Inflammation of Trinitrobenzenesulfonic Acid-Induced Rats and Suppresses P38 and JNK Pathways in Caco-2 Cells. https://pubmed.ncbi.nlm.nih.gov/25729217/.

[84] Shi L., Lin Q., Li X. (2017). Alliin, a garlic organosulfur compound, ameliorates gut inflammation through MAPK‐NF‐*κ*B/AP‐1/STAT‐1 inactivation and PPAR‐*γ* activation. *Molecular Nutrition & Food Research*.

[85] Kay H. Y., Won Yang J., Kim T. H. (2010). Ajoene, a stable garlic by-product, has an antioxidant effect through Nrf2-mediatedglutamate-cysteine ligase induction in HepG2 cells and primary hepatocytes. *Journal of Nutrition*.

[86] Joo H., Kim C. T., Kim I. H., Kim Y. (2013). Anti-obesity effects of hot water extract and high hydrostatic pressure extract of garlic in rats fed a high-fat diet. *Food and Chemical Toxicology*.

[87] Eslamparast T., Poustchi H., Zamani F., Sharafkhah M., Malekzadeh R., Hekmatdoost A. (2014). Synbiotic supplementation in nonalcoholic fatty liver disease: a randomized, double-blind, placebo-controlled pilot study. *American Journal of Clinical Nutrition*.

[88] Wigg A. J. (2001). The role of small intestinal bacterial overgrowth, intestinal permeability, endotoxaemia, and tumour necrosis factor *α* in the pathogenesis of non-alcoholic steatohepatitis. *Gut*.

[89] Mouzaki M., Comelli E. M., Arendt B. M. (2013). Intestinal microbiota in patients with nonalcoholic fatty liver disease. *Hepatology*.

[90] Berná G., Romero Gomez M. (2020). The role of nutrition in non‐alcoholic fatty liver disease: pathophysiology and management. *Liver International*.

[91] Ferro D., Baratta F., Pastori D. (2020). New insights into the pathogenesis of non-alcoholic fatty liver disease: gut-derived lipopolysaccharides and oxidative stress. *Nutrients*.

[92] Huang T. D., Behary J., Zekry A. (2020). Non‐alcoholic fatty liver disease: a review of epidemiology, risk factors, diagnosis and management. *Internal Medicine Journal*.

[93] Ried K. (2020). Garlic lowers blood pressure in hypertensive subjects, improves arterial stiffness and gut microbiota: a review and meta-analysis. *Experimental and Therapeutic Medicine*.

[94] Chen K., Xie K., Liu Z. (2019). Preventive effects and mechanisms of garlic on dyslipidemia and gut microbiome dysbiosis. *Nutrients*.

[95] Leung H. H., Yau Y. F., Leung K. S. (2019). Garlic supplementation modified enzymatic omega‐6 polyunsaturated fatty acid oxidation in mild hypercholesterolemia. *European Journal of Lipid Science and Technology*.

