# Supplementary material for: Therapeutic Effects and Mechanisms of Action of Garlic (Allium sativum) on Nonalcoholic Fatty Liver Disease: A Comprehensive Systematic Literature Review
Source: Evid Based Complement Alternat Med. 2022 Oct 6;2022:6960211. doi: 10.1155/2022/6960211 (PMC10292950; doi:10.1155/2022/6960211)
Supplement: Supplementary Materials — Supplementary Table 1. Search strategies including the key terms and the queries for each database. [file 6960211.f1.docx]

| **Supplementary Table 1.** Search strategies including the key terms and the queries for each database | |
| --- | --- |
| **Database** | **key terms and the queries** |
| **PubMed** | (“garlic” [MESH] or “ raw garlic” [TIAB] or “*allium sativum*” [MESH] or “fermented garlic” [TIAB] or “non-fermented garlic” [TIAB] or “black garlic” [MESH] or “diallyl disulfide” [TIAB]) and (“non-alcoholic fatty liver disease” [MESH] or “fatty liver” [TIAB] or “NAFLD” [MESH] or “liver fibrosis” [TIAB] or “NASH” [TIAB] or “non-alcoholic steatohepatitis” [TIAB] or “hepatic steatosis” [TIAB] or “insulin resistance” [TIAB] or “inflammation” [MESH] or “oxidative stress” [MESH] or “obesity” [MESH] or “BMI” [TIAB], “fat mass” [TIAB] or “dyslipidaemia” [TIAB] or “free fatty acids” [TIAB]) or “Glycaemic indices ” [MESH]. |
| **Web of Science (ISI)** | #1 TOPIC:  TOPIC: (" garlic”) OR TOPIC: (" raw garlic ") OR TOPIC: (" *allium sativum* ") OR TOPIC: (" fermented garlic e ") OR TOPIC: (" non-fermented garlic ") OR TOPIC: (" black garlic ") OR TOPIC: (" diallyl disulfide ")  #2 TOPIC: (" non-alcoholic fatty liver disease") OR TOPIC: ("fatty liver") OR TOPIC: ("NAFLD") OR TOPIC: (" liver fibrosis ") OR TOPIC: ("NASH ") OR TOPIC: (" non-alcoholic steatohepatitis ") OR TOPIC: ("hepatic steatosis ") OR TOPIC: (" insulin resistance ").  #3 TOPIC: (" inflammation”) OR TOPIC: (" oxidative stress ") OR TOPIC: (" obesity ") OR TOPIC: (" BMI ") OR TOPIC: (" fat mass ") OR TOPIC: (" dyslipidaemia ") OR TOPIC: (" free fatty acids ") OR TOPIC: (" Glycaemic indices ").  1 AND 2 AND 3 AND |
| **Scopus** | #1 TITLE-ABS-KEY (" garlic”) OR TITLE-ABS-KEY (" raw garlic ") OR TITLE-ABS-KEY: (" *allium sativum* ") OR TITLE-ABS-KEY (" fermented garlic e ") OR TITLE-ABS-KEY (" non-fermented garlic ") OR TITLE-ABS-KEY (" black garlic ") OR TITLE-ABS-KEY (" diallyl disulfide ").  #2 TITLE-ABS-KEY: (" non-alcoholic fatty liver disease") OR TITLE-ABS-KEY ("fatty liver") OR TITLE-ABS-KEY ("NAFLD") OR TITLE-ABS-KEY: (" liver fibrosis ") OR TITLE-ABS-KEY ("NASH ") OR TITLE-ABS-KEY (" non-alcoholic steatohepatitis ") OR TITLE-ABS-KEY ("hepatic steatosis ") OR TITLE-ABS-KEY (" insulin resistance ").  #3 TITLE-ABS-KEY: ("inflammation”) OR TITLE-ABS-KEY: (" oxidative stress ") OR TITLE-ABS-KEY: (" obesity ") OR TITLE-ABS-KEY: ("BMI ") OR TITLE-ABS-KEY: ("fat mass ") OR TITLE-ABS-KEY: ("dyslipidemia ") OR TITLE-ABS-KEY: (" free fatty acids ") OR TITLE-ABS-KEY: (" Glycaemic indices ").  1 AND 2 AND 3 AND |
| Embase | garlic /exp/mj OR raw garlic /mj OR' *allium sativum* '/exp/mj OR 'fermented garlic '/mj OR ' NASH '/exp/mj OR ' black garlic '/mj OR ' diallyl disulfide '/exp/mj  AND  'non-alcoholic fatty liver disease' /exp/mj OR fatty liver /mj OR ' NAFLD '/exp/mj OR 'liver fibrosis'/mj OR  ' non-fermented garlic '/exp/mj OR ' non-alcoholic steatohepatitis '/mj OR ' hepatic steatosis '/exp/mj OR 'insulin resistance'/exp/mj  AND  ' inflammation ' /exp/mj  AND  ' Oxidative stress' /exp/mj  AND  ' obesity ' /exp/mj  AND  ' BMI ' /exp/mj  AND  ' fat mass ' /exp/mj  AND  'dyslipidaemia' /exp/mj  AND  ' free fatty acids ' /exp/mj  AND  ' Glycaemic indices' /exp/mj |
| **Cochrane** | (1# MESH garlic or 2# TIAB raw garlic or 3# MESH *allium sativum* or 4# TIAB fermented garlic or 5# TIAB non-fermented garlic or 6# MESH black garlic or 7# TIAB diallyl disulphide in keywords  AND 8# MESH non-alcoholic fatty liver disease or 9# TIAB fatty liver or 10# MESH NAFLD or 11# TIAB liver fibrosis or 12 # TIAB NASH or 13# TIAB non-alcoholic steatohepatitis or 14# TIAB hepatic steatosis or 15# TIAB insulin resistance in keywords.  AND 16# MESH inflammation or 17# MESH oxidative stress or 18# MESH obesity or 19 #TIAB BMI, 20# TIAB fat mass or 21# TIAB dyslipidaemia or 22# TIAB free fatty acids) or 23# MESH Glycaemic indices in keywords. |
